# Influence of FFF Process Conditions on the Thermal, Mechanical, and Rheological Properties of Poly(hydroxybutyrate-co-hydroxy Hexanoate)

**DOI:** 10.3390/polym15081817

**Published:** 2023-04-07

**Authors:** Maria Rosaria Caputo, Mercedes Fernández, Robert Aguirresarobe, Adriana Kovalcik, Haritz Sardon, María Virginia Candal, Alejandro J. Müller

**Affiliations:** 1 POLYMAT and Department of Polymers and Advanced Materials: Physics, Chemistry and Technology, Faculty of Chemistry, University of the Basque Country UPV/EHU, Paseo Manuel de Lardizabal 3, 20018 Donostia-San Sebastián, Spain; 2 Department of Food Chemistry and Biotechnology, Faculty of Chemistry, Brno University of Technology, Purkynova 118, 612 00 Brno, Czech Republic; 3 School of Engineering, Science and Technology, Valencian International University (VIU), 46002 Valencia, Spain; 4 IKERBASQUE, Basque Foundation for Science, Plaza Euskadi 5, 48009 Bilbao, Spain

**Keywords:** additive manufacturing, poly(hydroxybutyrate-co-hydoxyhexanoate), biodegradable, mechanical properties, crystallinity

## Abstract

Polyhydroxyalkanoates are natural polyesters synthesized by microorganisms and bacteria. Due to their properties, they have been proposed as substitutes for petroleum derivatives. This work studies how the printing conditions employed in fuse filament fabrication (FFF) affect the properties of poly(hydroxybutyrate-co-hydroxy hexanoate) or PHBH. Firstly, rheological results predicted the printability of PHBH, which was successfully realized. Unlike what usually happens in FFF manufacturing or several semi-crystalline polymers, it was observed that the crystallization of PHBH occurs isothermally after deposition on the bed and not during the non-isothermal cooling stage, according to calorimetric measurements. A computational simulation of the temperature profile during the printing process was conducted to confirm this behavior, and the results support this hypothesis. Through the analysis of mechanical properties, it was shown that the nozzle and bed temperature increase improved the mechanical properties, reducing the void formation and improving interlayer adhesion, as shown by SEM. Intermediate printing velocities produced the best mechanical properties.

## 1. Introduction

Plastics are the most important materials for producing useful objects and molded parts in modern life. They are lightweight, have good mechanical properties, low corrosion properties, relatively low cost, and are versatile. However, many parts made of plastic materials are manufactured for single use. These materials take many years to degrade and contribute to the high volume of waste generated in the world (the so-called islands of plastic in the ocean, made up of microplastics of different origins), which is a severe environmental problem that exists today and needs to be solved. 

Many solutions to plastic pollution could be applied: (a) reduce the use of single-use plastics, (b) participate in a beach cleanup, (c) fulfill support legislation related to plastic waste, (d) recycle/reuse, and (e) make the public aware of this problem. Another solution is the development and use of more environmentally friendly materials. Many researchers are studying the use of biopolymers to be scaled in industry, such as poly(lactic acid) (PLA), poly(ε-caprolactone), poly(butylene succinate), poly(hydroxy alkanoates) (PHA), thermoplastic starch, among others. The production of these materials has been experiencing continuous growth in recent years, although it still represents less than 1% of the production of conventional plastics [1]. 

Polyhydroxyalkanoates (PHA) are linear thermoplastic polyesters of hydroxy alkanoic acids synthesized by various microorganisms and bacteria [2,3,4,5,6]. They have been proposed to replace some petrochemical-derived plastics [2,3,4], References [7,8,9,10] such as polyethylene, polypropylene, and polyethylene terephthalate. They are biocompatible, biodegradable, and non-toxic polymers that can be produced from renewable resources. They are highly crystalline, piezoelectric, and non-soluble in water.

PHA is considered one of the most important candidates to decrease the problem of plastic contamination [11], thus reducing the carbon footprint and contributing to a circular economy. However, PHAs have some disadvantages, including poor thermal-mechanical properties, susceptibility to thermal degradation, difficulty processing using conventional plastic processing techniques, and high production cost [12,13].

PHAs have a wide range of properties depending on the monomeric composition of polymers or copolymers, which are likely responsible for their different applications in various industries such as: (a) Biomedical sector: stents and artificial heart valves [14,15], pericardial patches, tissue engineering [16,17], nerve repair and regeneration [18,19,20], articular cartilage and tendon repair devices, bio-implant patches [21], sutures, tacks, staples, surgery [22], wound dressing [23], adhesion barriers, ocular cell implants, skin substitutes, prosthetics [24], meniscus repair devices, bone plates and bone plating systems, orthopedic pins, spinal fusion cages, bone graft substitutes, bone dowels, bone marrow scaffolds [25,26,27,28,29]; (b) pharmaceutical industries: biosurfactants and drug delivery systems [30,31,32,33,34,35,36,37,38]; (c) packaging sector (films, bags, containers, paper coatings) [39,40]; (d) disposable products (razors, cosmetic containers (shampoo bottles and cups utensils), diapers, feminine hygiene products [41]; (e) water treatment; (f) paper modification (sizing of paper); (g) cosmetic industries and (h) agricultural sector [42].

There are different PHAs produced at an industrial scale. These include poly(3-hydroxybutyrate) (PHB), poly(3-hydroxybutyrate-co-3-hydroxyvalerate) (PHBV), poly(3-hydroxybutyrate-co-4-hydroxybutyrate) (P3HB4HB), and poly(3-hydroxybutyrate-co-3-hydroxyhexanoate) (PHBH) [41,43,44]. 

The random copolymer PHBH shows a broader processing window than PHB and PHBV. Moreover, PHBH has good thermo-mechanical and physicochemical properties due to its tailorable composition of elastomeric (3-HH) and highly crystalline (3-HB) units [45].

Nowadays, there is a great interest in using this eco-friendly material (PHBH) for two applications: packaging (disposable bags, food packaging, and agricultural mulch films) [46] and tissue engineering (scaffolds) due to its flexibility and room temperature compostability, biocompatibility, and biodegradation properties. Specifically, the manufacture of scaffolds used in tissue engineering with PHBH is recommended through additive manufacturing (AM) (3D printing), more specifically, fused filament fabrication (FFF). Various PHA-based materials have already been processed with this technique: for example, Wu et al. studied the printability of esterified PHBV containing different fillers [47,48,49]. Furthermore, Tian et al. [50] have studied how the presence of wood flour improves the stiffness of PHAs and reduces the costs associated with their production. The use of the PHBH for biomedical applications is promising because it exhibits excellent mechanical properties, does not have cytotoxicity, and has a significant proliferation of mouse embryonic fibroblast cells. Additionally, its hydrolytic degradation is faster than PLA [51,52], and some blends with PHBH are used by FFF to improve specific properties.

Stanzani et al. [53] and Giubilini et al. [54] used PHBH reinforced with cellulose nanocrystals (CNCs) to make scaffolds for eco-sustainable regenerative medicine because of the increase in the degree of disintegration of the polymers under simulated composting conditions. Furthermore, FFF allows complex structures to be produced as scaffolds. Valentini et al. [55] studied the properties of the composite of fibrillated nano cellulose (NCF)/PHBH in 3D printing by FFF. The stress at break and elongation at break showed a maximum at 0.5 wt% NCF, but the presence of NCF did not affect the thermal degradation behavior of the polymer.

Kovalcik et al. [51] studied the properties of 3D printing gelatin-coated and non-coated scaffolds of PHBH. The gelatin-coated in the PHBH scaffold does not significantly affect the adhesion and proliferation of cells compared with the pure PHBH, which promotes cell growth due to its hydrophilicity.

However, no publications are reported in the literature on measuring the tensile properties of parts manufactured by FFF with PHBH and their relationship with thermal and rheological properties. This paper studies the relationship between the FFF processing conditions and the thermal, mechanical, and rheological properties, complemented by numerical simulation, to understand the process of deposition and cooling of the layers during PHBH additive manufacturing. The correlation between crystallinity degree, melt viscosity, and tensile test properties on printing conditions are presented. The mechanical properties of manufactured parts have been compared with compression-molded specimens.

## 2. Materials and Methods

### 2.1. Materials

A commercial thermoplastic biodegradable grade of poly(hydroxybutyrate-co-hydoxyhexanoate) with 6% hexanoate, PHBH, in pellets was employed. The PHBH denoted Green Planet™ X131A with a density of 1.2 g cm^−1^ and purchased from Kaneka Corporation (KITA-KU Osaka, Japan) was used. In a previous study by some of us [51], properties such as molecular weight (M_n_ number-average and M_w_ weight-average), polydispersity (Đ), and melt volume rates were determined, and they are reported in Table 1. 

### 2.2. Rheological Characterization

The rheological properties were determined using a strain-controlled ARES-G2 rotational rheometer (TA Instruments, New Castle, DE, USA). Samples of 1 mm thickness and 25 mm diameter were analyzed in parallel plate geometry. To minimize degradation effects, residual moisture was removed by drying the PHBH pellets overnight under vacuum at T = 60 °C, and rheometer experiments were performed under a nitrogen atmosphere.

Characterization included small amplitude oscillatory shear (SAOS) and continuous flow experiments. Viscoelastic functions such as elastic modulus, *G*^′^, viscous modulus, *G*^″^, and complex viscosity, η*, were measured in the linear viscoelastic regime (strain amplitude below 5%) in a frequency range from 628 to 0.628 rad/s, at varying temperatures from 130 to 180 °C. Two consecutive tests were performed at each temperature to check reproducibility, with each test lasting 3 min; the error between measurements was less than 4%. The time-temperature superposition (TTS) principle was used to shift frequency data into a single master curve at T = 190 °C. Continuous flow measurements at T = 190 °C were also carried out to test the validity of the Cox–Merz rule [56].

(1) $$\eta^{*}\left(\omega \right)\equiv {\left.\eta (\dot{\gamma )}\right|}_{\omega =\dot{\gamma }}$$

where $\eta^{*}$(ω) is the complex viscosity as a function of frequency and $\eta (\dot{\gamma )}$ is the viscosity obtained in continuous flow at the corresponding shear rates$\;\dot{\left(\gamma \right)}$. 

### 2.3. 3D Printing of the Samples

The PHBH X131A pellets were dried at 80 °C for at least 6 h. Filaments were prepared by extrusion of dried PHBH pellets at 150 °C at 20 rpm using a FilaFab PRO 350 extruder (D3D Innovations Limited, London, UK). The average diameter of the filaments was 1.75 ± 0.03 mm (measured with a digital caliper at several places) [51]. 

A model of the parts was designed and converted to STL file format for FFF. Then, the PHBH filament was printed using the TUMAKER Voladora V1 FFF machine, provided by Tumaker (Gipuzkoa, Spain), and controlled with the Simplify3D V5 Software (Simplify 3D, Cincinnati, Ohio, USA). The software was used to generate a G-code and then to set up the different processing conditions used in this study. The maximum printing size of the 3D printer was 22 × 22 × 30 cm (length, width, and height, respectively) with a nozzle diameter of 0.4 mm. In addition, a representative model of the logo of the University of the Basque Country was also printed to verify the printability of the material. 

### 2.4. Printing Conditions

Before manufacturing the samples, the filament was placed in an oven at 60 °C for 12 h to eliminate any trace of moisture. Different combinations of variables were used to find adequate printing conditions. Nine printing conditions were employed, which are shown in Table 2. This study used three factor levels (low (L), medium (M), and high (H)) and three process conditions (nozzle temperature (°C), bed temperature (°C), and printing velocity (mm/s)), employing the Taguchi experimental design method. The selected response variables to optimize were the Young modulus, tensile strength, and strain at break.

There are few works in the literature that report attempts at additive manufacturing by FFF for neat PHBH, so the levels of each printing parameter were specified based on the processing temperatures values for the materials used by Giubilini et al. [50] and Kovalcik et al. [48] for the PHBH scaffold preparation. These variables were modified to obtain adequate printing conditions to improve the mechanical properties. 

Samples were printed using the eight conditions of Table 2 in a rectilinear form of 45°/−45°. Once the adequate condition was determined (M12), specimens were printed in which the layers were all oriented in the same direction (that of the longest axis of the specimen) to mimic patterns obtained by compression molding, as reported by Candal et al. [57]. They molded PBS-based materials in this direction, obtaining mechanical parameters similar to those of injection molding. In all the conditions, the layer height and the fill density were 0.3 mm and 100%, respectively. Five specimens for each condition were printed. 

**Figure 1:**
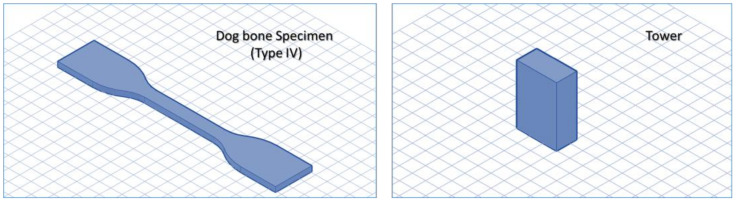
Parts/orientation printed in this work.

In this work, two different types of shapes were printed. The first ones were dog bone specimens (Type IV) with a flat-on configuration which were subsequently used to perform tensile tests (see Figure 1, left). The second type of specimen was a kind of tower in upright orientation (0.9 × 0.6 × 0.3 mm) to study the thermal properties of the different layers (Figure 1, right). 

### 2.5. Thermal Characterization of the Polymer

#### 2.5.1. Differential Scanning Calorimetry (DSC)

The thermal properties of the PHBH filament were measured. A TA Instruments Q2000 DSC calibrated with indium and tin under 50 mL/min of nitrogen flow was used to carry out these experiments. The samples were encapsulated in aluminum pans and were heated and cooled between −20 °C and 180 °C at 20 °C/min. The melting and crystallization temperatures and related enthalpies were measured, and from the fusion enthalpy, the degree of crystallization was calculated with the following equation:

(2) $$x_{c}=\frac{\Delta H_{m}}{\Delta H_{m}^{0}}\times 100\%$$

where $\Delta H_{m}$ (J/g) is the melting enthalpy of the samples and $\Delta H_{m}^{0}$ is the equilibrium melting enthalpy (122 J/g) calculated according to the group contribution method [58], as there are no experimentally extrapolated values reported in the literature for PHBH, but only for PHB. 

A Perkin Elmer DSC 8500 equipped with an Intracooler III as a cooling system was used to determine the melting temperature of samples taken in 5 different points of the 3D printed tower: in the first layer closest to the print bed, in the last printed layer, and in three intermediate layers. The experiments were performed under ultra-pure nitrogen flow using aluminum pans with around 7 mg samples. The equipment was calibrated with indium and tin as standards. The samples were heated from room temperature to 180 °C at 20 °C/min to explore differences in the melting points and related enthalpies.

#### 2.5.2. Pressure–Volume–Temperature (PVT) Measurements

PVT measurements were carried out using a PVT apparatus of the piston die type, PVT100, made by Haake. The sample was contained in a floating measurement cylinder (8 mm diameter), and pressure was applied hydraulically to a piston at the top of the cell with a PTFE disc seal. An identical piston and sealing system was located at the bottom of the cell. The data were obtained using an isobaric cooling mode procedure in a pressure range from 200 to 1000 bar with a cooling rate of 5 °C min^−1^, and the temperature range from 200 °C to 0 °C was controlled using liquid nitrogen. The results at a pressure of 1 bar were obtained by extrapolation to the Tait model included in the software [59].

The following equations give the 2-domain Tait PVT equation:

(3) $$v\left(T,P\right)=v_{0}\left[1-C\ln\left(1+\frac{P}{B\left(t\right)}\right)\right]+v_{t}\left(T,P\right)$$

where for polymers in the molten state, above the liquid-solid transition temperature:

(4) $$v_{0}=b_{1m}+b_{2m}\left(T-b_{5}\right)$$

(5) $$B\left(T\right)=b_{3m}exp\left[-b_{4m}\left(T-b_{5}\right)\right]$$

(6) $$v_{t}\left(T,P\right)=0$$

and for polymers in the solid state, below the liquid-solid transition temperature:

(7) $$v_{0}=b_{1s}+b_{2s}\left(T-b_{5}\right)$$

(8) $$B\left(T\right)=b_{3s}exp\left[-b_{4s}\left(T-b_{5}\right)\right]$$

(9) $$v_{t}\left(T,P\right)=b_{7}\;exp\left\{\left[b_{8}\left(T-b_{5}\right)\right]-\left(b_{9}P\right)\right\}$$

The liquid-solid transition temperature, which is the glass transition temperature for amorphous polymers and the melting or crystallization temperature for semi-crystalline polymers, is calculated by:

(10) $$T_{t}\left(P\right)=b_{5}+b_{6}P$$

In these equations, v is the specific volume, the coefficient *C* is a constant equal to 0.0894, *B (T)* is the sensitivity to pressure of the material, $b_{1m}$ and $b_{1s}$ to $b_{4s}\;$ describe the dependence on pressure and temperature in the molten and solid states, respectively. $b_{5}\;\mathrm{and}\;b_{6}$ are parameters that describe the change of transition temperature with pressure, $b_{7}\;$to $b_{9\;}$are particular parameters of semi-crystalline polymers that describe the form of the state transition.

### 2.6. Compression Molding

Specimens of the same size as those of Type IV (dogbone specimens for tensile tests) obtained by 3D printing were produced by compression molding. The equipment used for the hot-pressing process was a Collin P200E hydraulic press (Ebersberg, Baviera, Germany). A certain amount of material was placed in a mold and between the plates of a press at 170 °C and 200 bar. Preheating without pressure (2 min), compression under pression (3 min), and cooling under pressure (6 min) were carried out to produce the specimens. 

### 2.7. Tensile Tests

To perform tensile tests, an INSTRON 5569 testing machine was used. This test was performed for all the 3D printed patterns and for the specimens obtained by compression molding. The tests were performed according to ASTM D638 guidelines [60]. Young’s modulus, tensile strength, and tensile strain at break were measured using 20 mm/min as cross-head speed and 65 mm as the distance between grips. This analysis compares the mechanical properties of the 3D printed samples with those obtained by compression molding.

### 2.8. Cross-Sectional Morphology

To observe the cross-section of the samples obtained by 3D printing, SEM analysis was conducted with a HITACHI TM3030Plus Tabletop Scanning Electron Microscope (SEM) at 15 kV. Before the observation, all the samples were cryogenically fractured in liquid nitrogen and gold-coated in an SC7620 Mini Sputter Coater (Quorum). The images of the specimens were captured with a digital camera with a resolution of 25 nm. For comparative purposes, the samples obtained by compression molding were also observed.

### 2.9. Simulation

Temperature profiles were simulated in two dimensions, solving the heat transfer equation described in (11) using the heat transfer module of COMSOL Multiphysics 5.6 software (COMSOL, Stocolm, Sweden) in the geometry described in the text.

(11) $$\rho c_{p}\frac{\partial T}{\partial t}-\nabla \cdot \left(k\nabla T\right)=Q$$

where ρ is the material density, *c_p_* is the heat capacity, and k is the thermal conductivity, respectively. The calculations were conducted for PHBH using experimentally determined parameters summarized in Table 3.

Density. Density measurements were performed using an electronic densitometer (Mirage SD-120 L), and n-butanol was used as the immersion liquid. Six impact specimens were weighed for each reported value, and the immersion liquid temperature was determined (with 0.1 °C precision). The measured value is 1.21648 g/cm^3^.

Thermal conductivity: The measurement of thermal conductivity was carried out with the aid of the Gottfert Rheograph 25 instrument, following the ASTM D5930 standard, in a cooling and heating process in the temperature range between 30 °C and 190 °C. This value is 0.170 W/m*K for low temperatures and 0.198 W/m*K for high temperatures.

Thermal capacity: The measurement of the thermal capacity was carried out with a DSC Q2000, supplied by TA instruments, calibrated with sapphire and indium. The thermal capacity was measured during cooling in the temperature range between 50/30 °C and 200/180 °C.

The following boundary conditions were established: room temperature: 25 °C, platform temperature: 30 °C, and printing temperature: 190 °C. Extra layers were added manually to analyze the effect of the number of deposited layers.

## 3. Results

### 3.1. Thermal Analysis

#### 3.1.1. Differential Scanning Calorimetry (DSC)

- Virgin material (filament)

**Figure 2:**
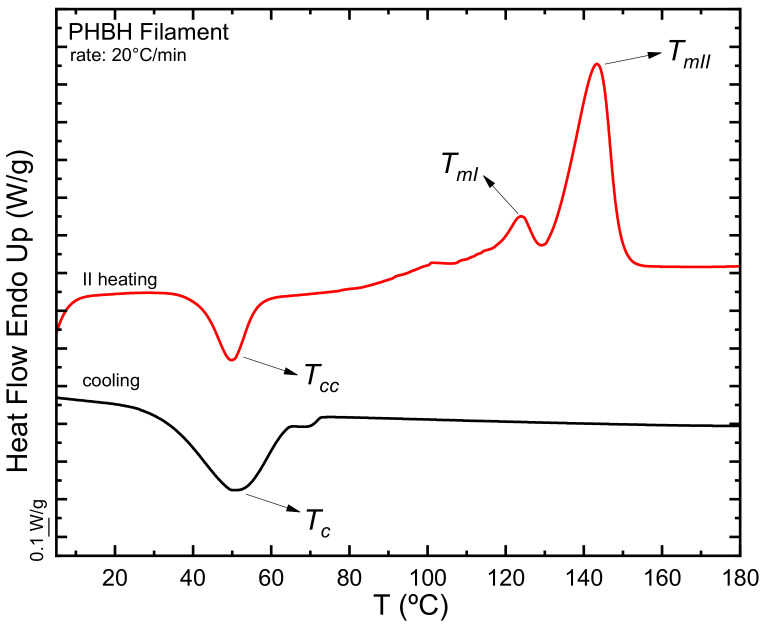
DSC scans of cooling and second heating for the filament of PHBH.

Figure 2 compares the cooling and second heating DSC scans of the PHBH filament. In the second heating, a cold crystallization phenomenon is observed in the sample, which then melts with a bimodal profile. Cold crystallization is a typical phenomenon of polyhydroxyalkanoates already reported by Caputo et al. [61] in the case of high molecular weight PHB. During cooling at 20 °C/min, the PHBH cannot complete crystallization until saturation; hence, it exhibits cold crystallization in the subsequent heating scan. The double melting peak observed is most probably due to melting and reorganization during the scan, as also reported for other thermoplastic materials [62,63]. However, a more detailed study (outside the scope of the present work) would be needed to confirm this fact.

Table 4 reports the thermal parameters obtained through these experiments. As expected, the crystallization and melting temperatures of PHBH and the related enthalpies, regardless of whether in the form of a filament or a 3D printed specimen, are lower than those reported for PHB [61], as the PHB is randomly copolymerized with the hydroxyhexanoate to obtain PHBH. The comonomeric units interrupt the crystallizable PHB sequences; therefore, the lamellar thickness is reduced and, concomitantly, the melting point. Analyzing these values made it possible to determine the minimum temperature for printing. Having to use a temperature at least 30 °C higher than the melting point to melt the polymer and erase its crystalline thermal history fully [64], the minimum temperature that could be used would be 170 °C. Despite this, at this temperature, the polymer does not reach such a fluidity to be extruded with the supplied printer used in this work. Therefore, the minimum printing temperature was set at 180 °C.

Furthermore, since the thermogravimetric analysis performed by Kovalcik et al. [51] showed that the degradation temperature of PHBH is 220 °C, the maximum printing temperature used in this work was 200 °C.

b. Printed part (Tower)

**Figure 3:**
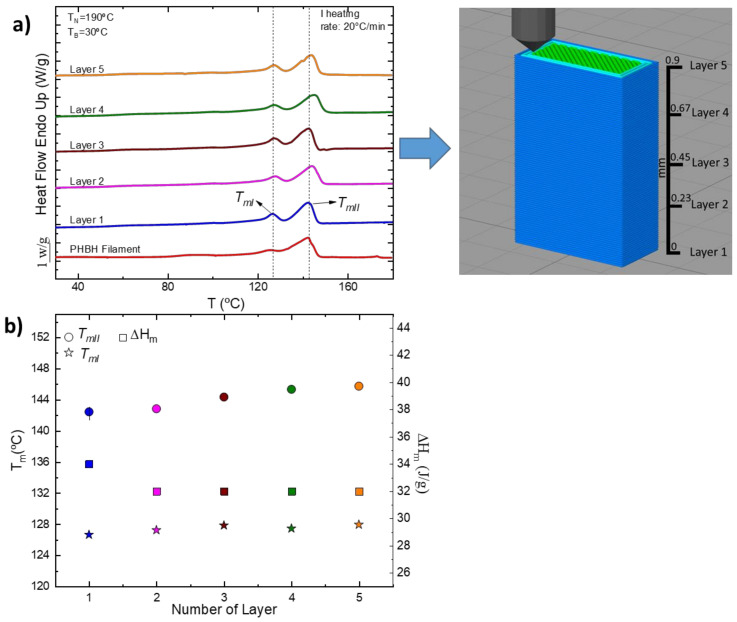
(**a**) First heating DSC scans of different layers of the PHBH tower and (**b**) melting temperatures as a function of the number of layers.

As described in Section 2.5.1, a study of the melting temperature of the different layers of a 3D-printed tower was carried out to observe any variations in this value and clarify whether the crystallization process occurs in an isothermal manner or not. A small sample was taken from five different layers in the tower, and a heating DSC ramp was performed. Figure 3a shows the DSC first heating scans for the five layers analyzed compared to the first heating scan of the filament. In Figure 3b, the melting temperatures and enthalpies are shown as a function of the layer number. As already found in the case of the filament, the scans of the various layers also show a bimodal melting peak; furthermore, only minor differences are observed in the melting temperatures and enthalpies values, as seen in Figure 3b. This result was unexpected as FFF is usually a non-isothermal process.

Because of the similar *T_m_* and Δ*H_m_* results, heat transfer simulations were carried out to better understand the thermal properties (crystallization conditions) during the 3D-printing process, specifically, why intrinsically non-isothermal manufacturing (FFF) leads to no differences in the crystallinity of the material. 

**Figure 4:**
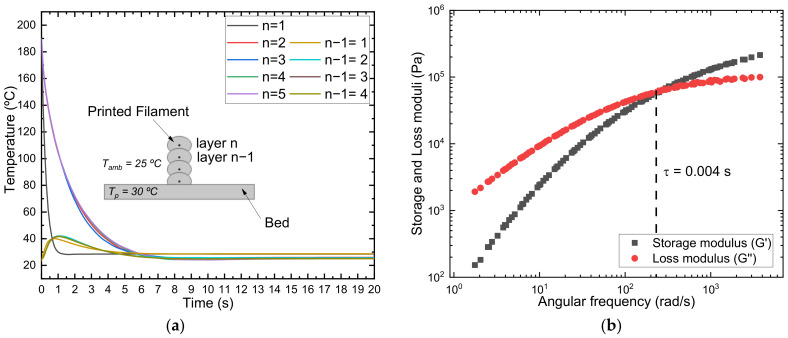
(**a**) Temperature profile during the cooling of the filament as a function of layer and (**b**) frequency sweep experiments at 190 °C. Relaxation time was calculated as the reciprocal of the cross-over frequency.

Figure 4a shows the temperature evolution for a printing system of different layers (layers 1 to 5). In that case, “n” represents the deposited layer, and “n − 1” represents the layer below the printed layer. According to the results, except for the first layer, the deposited polymer reached a constant temperature of 25 °C (*T_amb_*) in 5 s. This time is very short and, therefore, could prevent the crystallization of PHBH during the cooling from the melt. In addition, the temperature reached for the “n − 1” layer was around 40 °C, much below the cold crystallization temperature of the material. Although polymers are not good thermal conductors, the surface area in contact with air is sufficiently high to produce such a fast temperature decrease. In addition, the results are in good agreement with previously reported data for ABS [65]. More interestingly, the thermal profile that the material suffers during manufacturing is similar, regardless of the layer number, and therefore, no differences in crystallization should be expected among layers.

It is noteworthy that such a high cooling printing velocity might affect the interlayer adhesion of the printed specimens. However, as can be seen from the rheological frequency sweep experiments (Figure 4b), the relaxation time of the polymer chains at the nozzle temperature is below 10^−2^ seconds, which ensures interlayer adhesion [66]. 

#### 3.1.2. Rheological Characterization

The properties of biodegradable polymers are highly dependent on processing conditions such as humidity, temperature, shear rates, and processing time, so rheological characterization can provide valuable information for optimizing the 3D printing process of these materials.

Many efforts have been made to identify the relevant physical parameters that govern 3D printing. The application of rheological knowledge to understand the critical physics and implications in all aspects of the process, involving nozzle flow, the nozzle-bed standoff region, and finally, the deposition on the print bed, is considered of great importance to determine and identify the correlation between material properties, processing variables, and the resulting mechanical properties. Most recent reviews, gathering numerous studies and detailed discussions on the subject, highlight the following key aspects to deep in this understanding [67,68,69,70]: (a) temperature-dependent shear and extensional viscosity correlate with the extrusion quality through the print nozzle and in the region between the nozzle and the bed; (b) chain dynamics as the melt cools once deposited, governs the degree of interlayer welding and controls mechanical performance; (c) the evaluation of the flow-induced crystallization under complex flow and thermal fields developed within the nozzle is relevant in the predictions of the mechanical properties. Therefore, evaluating the polymer relaxation dynamics under the combined effect of shear rate and temperature profile, from the perspective of the disentanglement and orientation state of the chains during extrusion and post-extrusion entanglement recovery, is contemplated as essential for the improvement of the simulation and optimization of the 3D printing process.

The viscoelastic behavior of the polymer determines two essential aspects of the 3D printing process: the extrusion of the melt through the nozzle and the welding of layers during the subsequent deposition stage [57]. Thus, the analysis of the dynamics and time scales of the polymer and how they are affected by the printing temperature and velocity will be fundamental aspects of the rheological characterization performed in this work.

**Figure 5:**
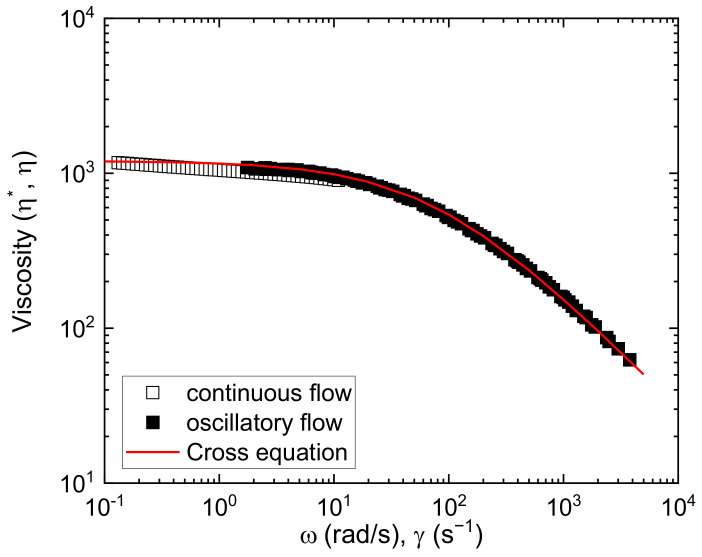
PHBH viscosity curves at T = 190 °C from continuous flow and oscillatory flow (TTS superposition master curve). A fitting to the Cross equation is included [*η*_0_ = 1200 Pa s, λ_0_ = 0.013 s, and α = 0.75].

The melt viscosity and its dependence on temperature and shear rate determine the ability of polymers to flow. Figure 5 shows the melt viscosity curves of PHBH at T = 190 °C. The aforementioned Cox–Merz rule is fulfilled as a good correlation of continuous values, $\eta (\dot{\gamma ),\;}$ and dynamic, $\eta^{*}\left(\omega \right),\;$viscosities are observed as a function of frequency. Considering that the rule does not hold for phase-separated (e.g., block copolymers) or complex polymer systems, this result suggests the random nature of the PHBH copolymer [56].

As can be seen in Figure 5, PHBH flow is quite sensitive to shear, the pseudoplastic behavior well characterized by the Cross equation [71]:

(12) $$\eta =\frac{\eta_{0}}{1+{\left(\dot{\gamma }\lambda_{0}\right)}^{\alpha }}$$

where $\eta_{0}\;$is the Newtonian viscosity, $\lambda_{0\;}$is the relaxation time, and α a non-linearity index. The values of the fit of experimental data to the equation for the polymer PHBH are included in Figure 5. The viscosity curve is comparable to commercial materials widely used in filament-based 3D printing. As an example, at typical extrusion shear rates of $\dot{\gamma }=$ 200 s^−1^, the viscosity of PHBH, *η* = 400 Pa s, lies between the viscosities of ABS acrylonitrile-styrene (ABS), *η* = 1000 Pa s, and that of polylactic acid (PLA), *η* = 200 Pa s, as observed in Figure 9 of [57] (Candal et al., 2020). Therefore, as a first result, the flow behavior of this biodegradable polymer would meet the requirements to be processed by extrusion during 3D printing.

However, the complete evaluation of this polymer requires special attention to its welding response, which is one of the critical parameters of 3D technology. Poor adhesion between deposited filaments often results in poor mechanical properties of the printed samples. Polymer-polymer welding, necessary to ensure the strength of the final printed part, implies a satisfactory interdiffusion and reentanglement of the molten polymer across the filament-filament interfaces [72]. Thus, this property, which is highly dependent on the viscoelastic response, will also be addressed in our study. 

The entanglement due to the hypothetical tubular region that restricts the diffusive motion of a polymer chain can be modeled to obtain the characteristics time scales that account for the molecular dynamics: reptation time, τ*_d_*, which governs chain orientation and alignment to relax, and the Rouse time τ_R_, which is the time for the polymer chain to relax within the tube region and governs stretch relaxation [73]. Under typical printing conditions, the residence time is similar to the reptation time, *τ_d_* (related to the time that has been determined with the Cross Equation,$\;\lambda_{0}$) and which allows assuming that a stationary flow develops at the printer nozzle. The relevant parameter that gathers information about the diffusion of the entanglement in the weld zone is considered to be the entanglement density, *Z_e_*, which is defined as *Z = M_e_*/*M_w_*, where *M_e_* is the molecular weight of the entanglement and *M_w_* is the molecular weight of the polymer. *M_e_* is related to the entanglement modulus, $G_{N\;}^{0}$ through the generally accepted equation:

(13) $$G_{N}^{0}=\rho RT/M_{e}$$

where ρ is the density, and *R* is the gas constant. 

**Figure 6:**
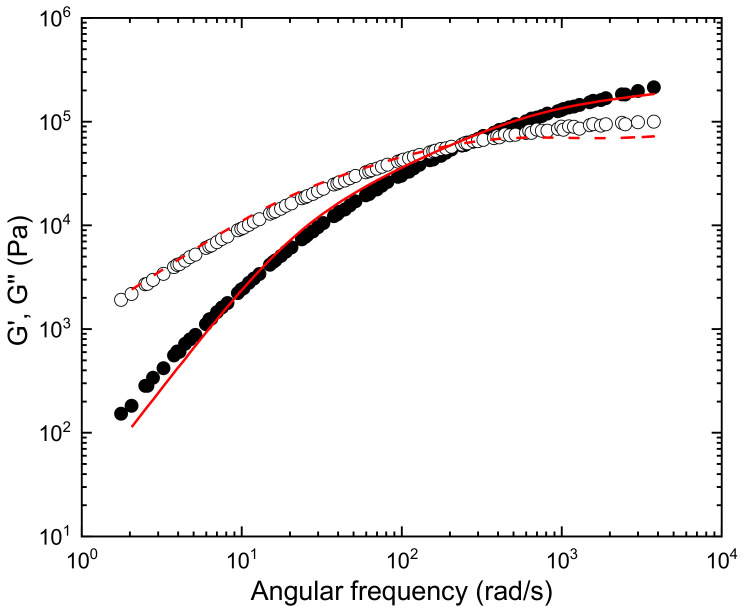
Master curve at the reference temperature T = 190 °C for PHBH fitted to the Likhtman–McLeish theory. [$G_{N\;}^{0}=400,000\;\mathrm{Pa},\;$*M_e_* = 8960 g/mol and τe = 3.5 × 10^−6^ s, τ*_d_* = 0.026 s, τ*_R_* = 0.001s].

The experimental viscoelastic functions, storage modulus, *G*^′^, and loss modulus, *G*^″^, measured at different frequencies and temperatures, were analyzed using the time-temperature superposition method. The nice superposition is shown in Figure 5 (complex viscosity) and Figure 6 (storage and loss moduli). Shift factors, *a_T_*, follow a temperature Arrhenius-like dependence, which is given by:

(14) $$a_{T}=Ae^{-}\frac{E_{a}}{RT}$$

where *A* is a pre-exponential factor, *R* is the gas constant and $E_{a}$ = 51 KJ/mol is the flow activation energy. The master curve obtained at T = 190 °C, as the reference temperature, contains information about the terminal and the rubbery zones, from which it is possible to calculate the entanglement density in the absence of shear, *Ze*. To determine this parameter, the experimental master curve was fitted to the Likhtman–McLeish theory [74] using RepTate software (open source) [75].

The entanglement molecular weight, *M_e_*, is then used to characterize the entanglement density, *Z_e_*. Considering the molecular weight of *M_w_* = 163,000 g/mol (Table 1), a value *Z* = 18 is obtained for PHBH.

According to the model of McIlroy and Olmsted [72], the entanglement number *Z_e_* should be in the range *Z* = 20–30 to obtain a good welding response. The lower limit is very close to the result estimated here for PHBH and similar to those found in the literature for printable PLA polymers with good interlayer adhesion, such as the value of *Z* = 17 for polylactic acid (PLA) where the mass *M_w_* = 156,000 g/mol and *M_e_* = 9000 g/mol [76], and the value of *Z* = 19 for the PLA polymer of *M_w_* = 173,000 g/mol and *M_e_* = 9000 g/mol [73]. Notwithstanding, an acceptable strength across the interface, with considerably lower Z values, was observed for some polymers, as in the case of PBS and PBSA [57]. In particular, a value of *Z* = 8 was reported for PBS with *M_e_* = 8750 and *M_w_* = 79,250, and a value of *Z* = 9 for PBSA with *M_e_* = 9000 and *M_w_* = 78,600. This leads us to consider that molecular chain diffusion might not be the only controlling factor during weld formation for these polymers.

In fact, the high speed of cooling inherent to the 3D printing process could also affect interlayer welding. The polymer chains have to diffuse across the layer interface at a distance equivalent to the radius of gyration (Rg) to ensure interlayer adhesion [72]. The extent of interdiffusion will depend on the time available before the chain loses mobility due to crystallization [77]. At the temperatures selected in this study Section 3.1.1, the PHBH chains are expected to have sufficient time for good interlayer adhesion to be acquired. The interdiffusion time of PHBH, in terms of the reptation time, τ*_d_* = 0.026 s (shown in Figure 6, T = 190 °C), is close to the value reported by Das et al. [77] for PLA, τ*_d_* = 0.048 s (T = 190 °C) subjected to a printing process characterized by bond strength increasing with printing temperature and crystallization that did not impede interdiffusion dynamics. Moreover, the time for welding could be even lower if models based on reptation adapted to non-isothermal conditions are considered. Yang et al. (2002) [78] studied PEEK-based polymers during 3D printing. They suggested that experimental welding times shorter than the reptation time could be explained if the interpenetration length at the weld is redefined. Indeed, minor chains (end chains) would not need to diffuse across a distance equal to Rg to reach bulk properties. 

The effects of molecular orientation during polymer extrusion must also be considered. Typical shear rates in 3D printing vary between 100 s^−1^ and 1000 s^−1^ in the non-Newtonian flow regime of polymers, where chains can be oriented and aligned in the direction of flow if the deformation is faster than molecular relaxation times. In case the macromolecular chain orientation can be retained after deposition, the resulting anisotropy could lead to improved mechanical properties such as elastic modulus and tensile strength, as reported by Gonzalez Ausejo et al. (2018) [79] for a PLA/PHA blend. A more ordered chain configuration can facilitate and enhance crystallization, as observed for PCL (Liu et al. (2018)) [80] and polyamide (PA) 12 (De Jager et al. (2020)) [81].

On the other hand, interlayer welding can also be affected. Models [73,82,83,84] predict that the development of oriented polymer chains results in a shear-induced disentanglement process that decreases entanglement densities. This could negatively impact welding energy for printing conditions where entanglement does not have sufficient time to recover during cooling, as the degree of chain stretching during shear is determined by the competition between shear deformation and chain relaxation in the tube. Printing at low temperatures and high velocities was found to decrease the weld strength in the prints of multiple semi-crystalline polymers, including PLA, PP, and PA [67]. However, the extrusion-induced orientation can also be relaxed after deposition with increasing build temperature or residence time because polymer chain mobility is enhanced, and thus relaxation can occur more easily as orientation gets lost. For a PLA with low crystallinity [73], the weld strength decrease is observed at printing velocities above 60 mm/s at lower temperatures, when relaxation times are expected to increase considerably. On the other hand, a small effect of printing velocity on tearing energy is reported for PBSA and PBS [57] because larger velocities than applied are probably needed to observe significant effects. This seems to be the case of the printed samples of PHBH studied here, where the good adhesion, evaluated in terms of homogeneity of the interlayers observed by SEM [Figure 10, shown below in Section 3.3], is not deteriorated by the printing velocity. Then, it can be assumed that the entanglement density is slightly affected by the shear rate or that the chains have enough time to relax and re-entangle. 

Certainly, the formation of welds during 3D printing is very complex. Although, as mentioned above, there is an apparent loss of entanglement with increasing alignment, the rate of chain reorientation would still be close to the equilibrium values given by the tube models of polymer dynamics, as discussed by Gunha et al. (2020) [85]. Thus, interfacial entanglement reformation should be relatively insensitive to alignment, and welds of oriented molecules should be as strong as bulk isotropic materials when the molecules have sufficient time to diffuse along their tube. This is certainly not the general behavior in 3D printing processes. With this concept in mind, Seppala et al. (2017) [86] analyzed the welding between ABS layers under rapidly changing mobility conditions. They discussed several factors that may contribute to the underperformance observed in 3D printing, including the necessity of longer effective welding times, degradation at higher temperatures, stress concentration due to the shape of the part, and the polymer alignment playing an important role. 

In summary, the welding might not be limited to inter diffusion, and other arguments should be considered. Similarly, Constanzo et al. (2022) [87] discussed the effect of anisotropy on welding. They analyzed the behavior of copolyesters with different relaxation times but similar welding properties. The different chain stiffness of these copolyesters affected the non-equilibrium configuration of the entanglement network after printing in a way that the lower molecular extensibility of the stiffer chains was related to a decrease in anisotropy degree able to regulate the weld response. The results agreed with the findings from molecular simulations of 3D print samples, where the residual molecular anisotropy could affect the mechanical properties since the aligned material near the weld is weaker than the non-aligned material [85]. Therefore, the alignment effect at high printing rates could decrease mechanical properties, even though the reptation times indicate sufficient time to diffuse along the tube.

It should also be noted that molecular dynamics and processing conditions could affect the crystallization dynamics, as stated before. The development of the oriented shear-induced crystallinity precursors depends on the deformation rate that is experienced by the polymer melt. The flow strength can be quantified by dimensionless numbers, such as the Deborah number, De, and the Weissenberg number, Wi. Following the analysis proposed by McIlroy and Olmsted [84,88], the description of the 3D printing process can result in flow-induced crystallization under the following conditions: (1) The flow is sufficiently strong to stretch polymer chains, a condition that can be quantified by the Rouse Weisenberg number ($W_{iR}=\dot{\gamma }\tau_{R}$) that governs polymer stretching, greater than 1. (2) When the residual stretch persists at the onset of nucleation, a condition that can be quantified by the inverse of the Deborah number, *D_e_^−1^*, defined as the ratio of the time taken for the material to cool to the melting temperature, *τ_m_*, to the stretch relaxation time, *τ_R_*, $De^{-1}=\frac{\tau_{m}}{\tau_{R}},\;$lower than 1. Under these conditions, $W_{iR}>1\wedge De^{-1}<1,$ the stretched polymer chains would provide flow-enhanced crystallization. The apparent shear rates considered in this study (8 *V*/*d*, where *V* is the printing velocity and *d* = 0.4 mm is the nozzle diameter) were of the order of $\dot{\gamma }=400\;s^{-1}$ (*V* = 20 mm/s) to $\dot{\gamma }=800s^{-1}$ (*V* = 40 mm/s). According to the Rouse/stretch relaxation time of PHBH, estimated by fitting viscoelastic moduli to the Likhtman–McLeish theory (*τ_R_* = 0.001 s, see Figure 6), the shear rate values are sufficiently low so that the applied flow does not stretch polymer chains. In addition, the condition of *De*^−1^ < 1 will be acquired only for *τ_m_* < 10^−3^ s, a value which is not expected for the 3D printing procedure considered here (see temperature profile in Figure 4). The analysis is of proven utility in the discussion of flow-enhanced structures during extrusion printing of polylactic acid (PLA) and polycaprolactone (PCL) [88]. PC analysis shows that at high temperatures, the polymer stretch becomes fully relaxed before the temperature reaches the melting point, so there is no flow-enhanced crystallization. However, the analyzed PLA behaves quite different because it has a much higher glass transition temperature that will arrest crystallization during printing. The entanglement time calculated for PHBH (τe = 3.5·10^−6^s), which is even lower than the corresponding value for PCL (τe = 1.9·10^−5^s), allows expecting a similar behavior for both polymers. As explained above, the *W_iR_* and *De*^−1^ calculations for PHBH predict that flow-induced crystallization is not favored under the printing conditions studied here.

#### 3.1.3. Evaluation of the Effect of Pressure and Temperature on Specific Volume/Density

The pressure-volume-temperature (PVT) diagram is widely used in science and industry for polymer injection molding [89]. However, the exact change of the specific volume during the printing process is usually not considered. Only a few recent papers refer to density models that simulate the velocity profile inside the nozzle and study the effect of pressure on viscosity and flow inside the printer [90,91].

In this work, following the perspective of investigating the parameters that affect the strength and quality of the printed parts, for example, from the rheology and microstructure, as already discussed in the rheological characterization section, we will try to introduce a qualitative analysis of the observed shrinkage and warpage in terms of the pressure-volume-temperature data obtained for the PHBH polymer.

**Figure 7:**
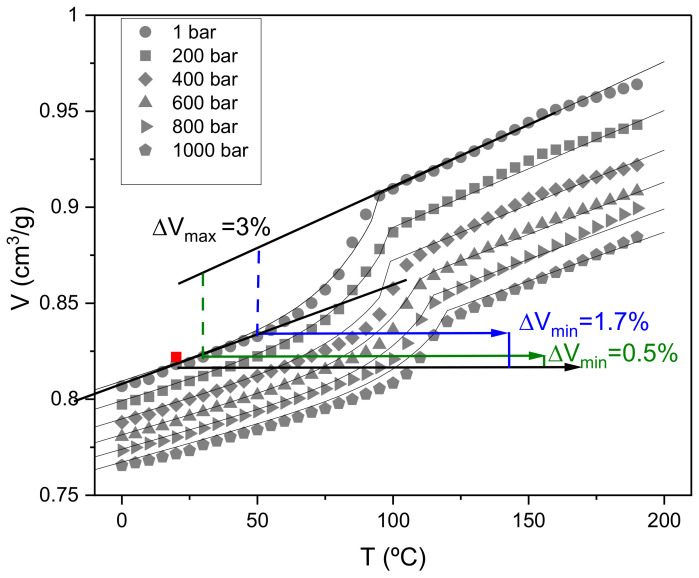
PVT diagram obtained under the isobaric cooling condition at 5 °C/min for PHBH polymer. Grey symbols are experimental-specific volumes at the pressures specified in the legend. Lines correspond to the Tait equation. The red symbol corresponds to the density of the printed part measured using an electronic densitometer (not PVT data). Changes in the specific volume are discussed in the text (lines in blue correspond to changes when *T_bed_* = 50 °C, and lines in green correspond to changes for *T_bed_* = 30 °C).

Figure 7 presents the PVT diagrams obtained under isobaric cooling for the PHBH polymer. The experimental data were fitted to the Tait equation, a two-domain empirical model to plot the specific volume as a function of the process variables: pressure and temperature (changes due to shear during extrusion are not considered). Fitting parameters are included in Table 5. 

**Figure 8:**
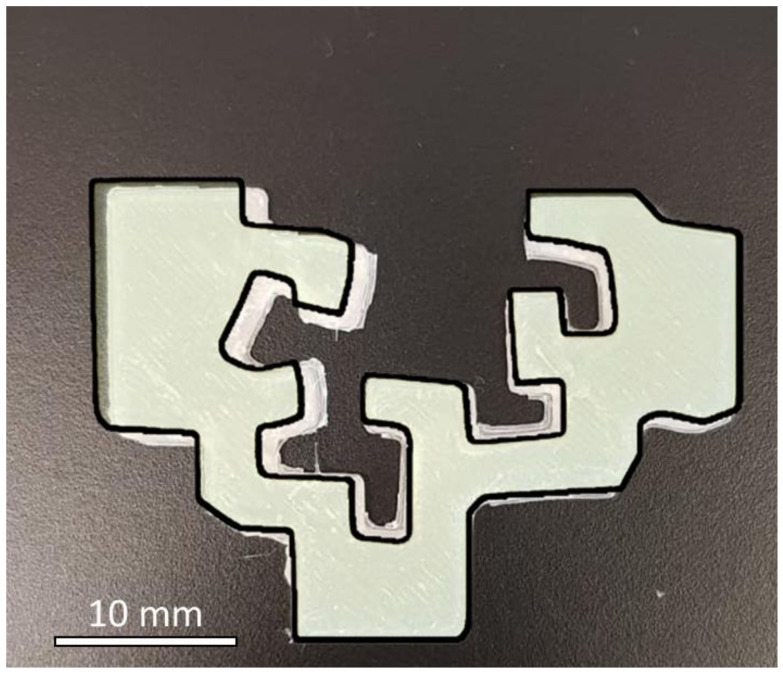
Image of the printed logo of the UPV/EHU with a dimensional comparison with the model.

Most extrusion processes are carried out in a narrow range of pressures, generally not exceeding 1000 bar, and density changes inside the nozzle must be accounted for by taking the density of the melt at that pressure. However, it has been reported that the value of the specific volume at the start of the process does not affect the final shrinkage. Therefore, in 3D printing, since filament deposition is performed at atmospheric pressure (1 bar) with temperature changing very rapidly from the extrusion temperature to the bed temperature, set at T = 30 °C or T = 50 °C, the most important volume change will be due to the crystallization process taken at 1 bar. In fact, during cooling, three different regions are characteristic: molten, transition, and solid zone. It is expected that during the solidification of the filament, the rapid cooling will shift the crystallization transition, in the diagram at *T_c_* = 100 °C, towards lower temperatures and that the main shrinkage taking place before the filament is deposited, as marked in Figure 7, would be due to crystallization (3.8%). Once the filament is deposited, the shrinkage is governed by the thermal expansion coefficient of the solid part, which accounts for the volume change calculated by taking the specific volume at bed temperature and the specific volume at room temperature (this would be the minimum shrinkage expected in the final printed piece). In the case of PHBH, as the Tg = 0 °C, this coefficient is still large enough to give rise to some shrinkage that materializes as minimal distortions of the part dimensions, as seen in Figure 8.

In addition, Figure 7 shows the specific volume of a sample obtained in the 3D printing process. The fast solidification process does not prevent crystallization as the density value is very similar to that obtained under slow solidification conditions, in agreement with the crystallinity values determined by DSC included in Figure 3. 

The PVT data at the exact temperature profile of the filament during deposition can give us information about the evolution of the shrinkage of the sample. The PVT diagram contains valuable information when the experimental procedure is designed correctly (hold temperature, cooling time) to investigate and optimize the reduction of dimensional distortions of 3D printed parts. 

### 3.2. Mechanical Properties

#### 3.2.1. Effect of the Bed and Nozzle Temperatures

To determine the best printing conditions of the PHBH, the discussion of the results obtained in the mechanical tests by varying the printing conditions is carried out in this section. Table 6 shows the mechanical parameters of Young’s modulus, tensile strength, and strain at the break of the 3D-printed specimens at 180 °C, 190 °C, and 200 °C, keeping the printing velocity constant (30 mm/s) and varying the bed temperature (30 °C and 50 °C). 

As can be seen, an increase in nozzle temperature leads to an improvement in mechanical parameters. Several authors reported similar results for other materials [92,93,94,95,96]. In all cases, this trend is attributable to the fact that high nozzle temperatures reduce the formation of voids because the viscosity of the resin and the air pressure decreases. 

In the case of the material under consideration in this work, the PHBH, it is possible to note from Table 5 that the variation of the nozzle temperature from 180 °C to 190 °C results in an increase of 9.7% of the tensile strength and 15% of the tensile strain at break. Although the increase is not as high as for the other materials reported in the literature, this could be due to the particular isothermal crystallization of PHBH during the printing process, confirmed by the simulation results, which leads to a printed pattern with few voids. This will be verified by the SEM analysis reported below.

By increasing the nozzle temperature to 200 °C, the tensile strength and tensile strain parameters remain constant within the errors reported in Table 5. This is probably attributable to the beginning of the degradation of the sample, as already mentioned previously and confirmed by thermogravimetric analysis. 

Moreover, it is known that for semi-crystalline polymers, a higher print bed temperature leads to an improvement in the strength of the interfacial bond and the dimensional accuracy of printed patterns due to the longer time for molecular diffusion before the onset of crystallization [71,72,97,98,99] This behavior has been reported for PP [100], and PLA [101]. In these cases, parts printed with high temperatures in the print bed can achieve mechanical properties comparable with specimens obtained by injection molding. Furthermore, this trend is also confirmed by Xiaoyong et al. [102] for PEEK, in which higher bed temperatures can improve the interfacial strength between layers in the printing process.

In the case of PHBH, as can be seen from the values reported in Table 5, a slight improvement in the tensile strength and tensile strain values is observed using a bed temperature of 50 °C.

#### 3.2.2. Effect of the Printing Velocity

Finally, Table 7 shows the mechanical parameter values obtained from printed specimens using 190 °C as the nozzle temperature and 50 °C as the bed temperature at different printing velocities. The values of the nozzle and bed temperatures were chosen based on the best results recorded in the specimens printed at velocities of 30 mm/s. As seen from Table 7, even the change in printing velocity determines a change in Young’s modulus and ductility of the specimens. 

A printing velocity of 20 mm/s produces specimens with Young’s modulus value of 23% lower than that found in specimens printed at velocities of 30 mm/s. A decrease in the toughness of the specimens is also observed: the values of tensile strength and strain at break decrease by 28% and 13%, respectively. This could be attributed to the fact that a slower print velocity allows the previous layers to crystallize before the next layer adheres to them because the heat dissipates rapidly [103]. This results in less compactness in the material and, therefore, poorer mechanical properties, as was also found in the case of PLA printed at low printing velocities [104] and PLA with wood fiber [105]. 

A lowering of mechanical performance is also found in specimens printed with a printing velocity of 40 mm/s, in which there is a decrease of 13% in the value of Young’s modulus and 10% in the value of tensile strength compared to the samples printed at 30 mm/s. This behavior could be explained by Abeykoon et al. [104]. They found that for PLA, a printing velocity higher than 90 mm/s could influence the fusion of the filament as polymers have poor thermal conductivity, leading to adhesion problems between the layers and, therefore, inferior mechanical properties. Thus, as in the case of PLA, it has been found that the best printing velocity is 90 mm/s; while the recommended print velocity is 30 mm/s for PHBH. This is also an advantage in terms of energy savings since higher printing velocity would lead to higher energy consumption and, because they do not result in an improvement in the mechanical properties, in this case, it is not worth using higher printing velocities. In terms of the final quality of the printed part, greater vibrations are generated with the highest printing velocity when the nozzle changes printing direction. Consequently, the printed polymer will have ringing or ghosting artifacts or even produce layer shifting during printing.

#### 3.2.3. Effect of the Raster Angle 

Table 8 compares the mechanical properties of the specimens obtained following the M12 conditions (190 °C as nozzle temperature, 30 °C as bed temperature, and 30 mm/s as printing velocity, but with different raster orientations) and the specimens obtained by compression molding. To mimic the compactness conditions obtained during compression molding, the raster orientation of the layers has been set in such a way that each layer is oriented in the same direction as the previous one, which is that of the longest axis of the specimen, corresponding to the direction at which the traction occurs.

According to the results obtained in this study, 3D printed samples with the layers oriented in the same direction to each other, which is also the direction of stretching [M12 (90°, 90°)], exhibit better properties than samples where the layers are oriented at 45° to each other [M12 (45°, 45°)]. Indeed, there is a 6% increase in Young’s modulus, 17% in tensile strength, and 30% in the strain at the break between the two types of printed samples. This behavior can be attributed to the fact that the layers are oriented in the same direction in which the stretching force is applied. Therefore, the sample could strengthen under stretching and oppose a higher resistance before breaking. Similar results were found in the case of materials based on ABS [106] and PP filled with short carbon fibers [107]. 

#### 3.2.4. FFF vs. Compression Molding

The specimens obtained by compression molding have a higher toughness than the 3D-printed specimens [90°, 90°] (with a 47% higher strain at break value) but maintain similar parameters of Young’s modulus and tensile strength. This is generally due to the presence of pores [108], anisotropy, and poor adhesive strength between the layers of the 3D printed pattern [109,110,111,112], which negatively affect the mechanical performance of the specimens. 

**Figure 9:**
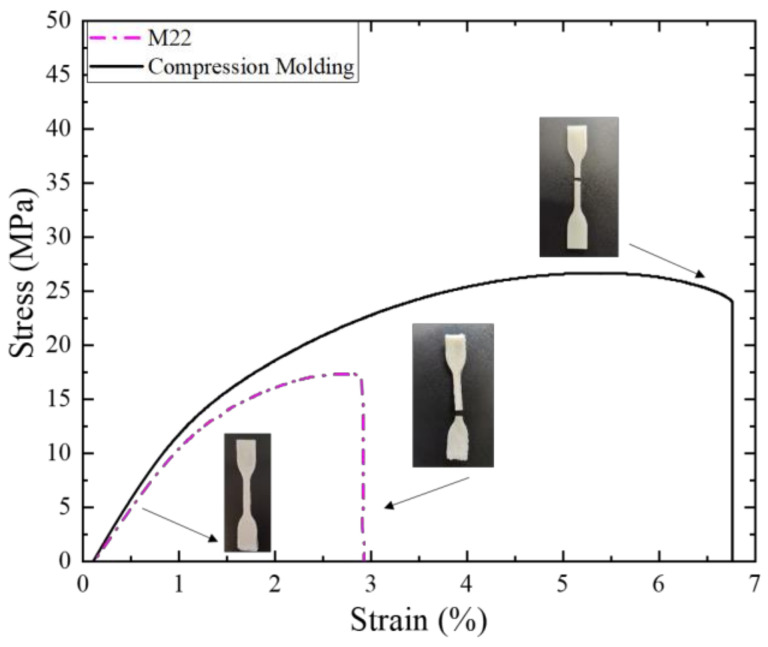
Tensile stress-strain curves of 3D printed using the M22 condition and compression molding PHBH.

Figure 9 shows the typical stress-strain curves of 3D-printed samples using the M22 condition listed in Table 2 and compression molding specimens. Photographs of representative 3D-printed specimens at the beginning and end of the test are also shown in the figure, and it is possible to appreciate the high quality (i.e., resolution) of the specimens. Both specimens have a brittle behavior characterized by breaking before reaching the yielding point.

### 3.3. SEM Analysis: Cross-Sectional Morphology

**Figure 10:**
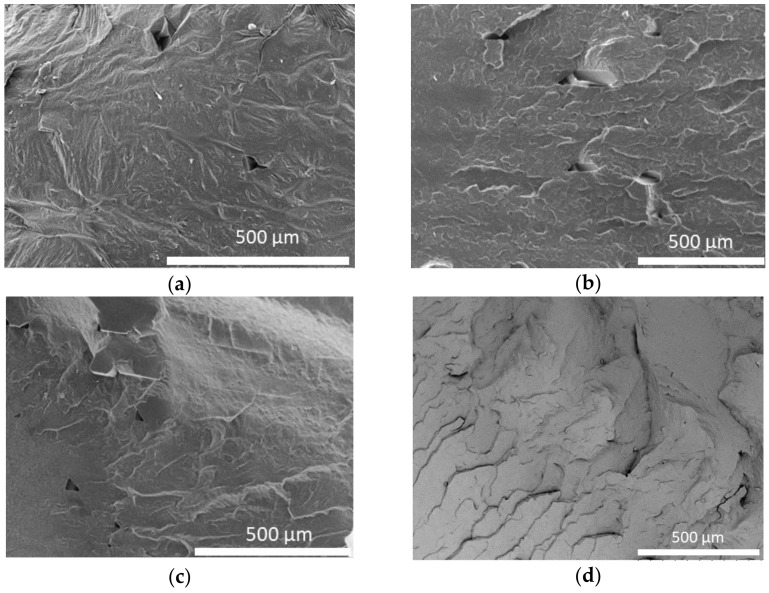
Micrograph of the cross-section of printed filaments obtained employing L22 (**a**), M22 (**b**), M12 [90°, 90°] (**c**), and compression molding (**d**).

Figure 10 shows SEM micrographs of the cross-section of the 3D printed specimens obtained with different conditions (a, b, and c) and compression molding specimen (d). No differences in the morphology are observed depending on the printing conditions; consequently, changes in the parameters of the printing process do not affect the morphology. The samples’ morphology appears compact, indicating that during the printing process, the adhesion between the different layers took place efficiently, thus avoiding the formation of many air gaps. As expected, the only difference between the specimens manufactured by FFF and those manufactured by compression molding was that no trapped air nor any holes resulting from 3D printing were observed in the specimens obtained by compression molding.

This behavior is different from that previously reported for PBSA by Candal et al. [57], in which it was possible to distinguish the various filaments in the layers of the printed pattern. In contrast, a similar behavior was reported by Abeykoon et al. [104] for PLA, for which 3D-printed patterns with 100% infill density have no air gaps. The behavior is consistent with the different entanglement densities calculated for these samples, as indicated in the rheological section. The entanglement density for the PBS (*Z* = 8) and PBSA (*Z* = 9), considerably lower than the density calculated for PHBH (*Z* = 18) and PLA (*Z* = 19), could explain the different welding between polymer layers. 

## 4. Conclusions

In this work, a comprehensive study of the printing properties of PHBH was conducted. DSC results on 3D printed specimens showed that, in the case of PHBH, unlike what usually happens in the FFF field, the crystallization during the printing process occurs in an isothermal manner after layer deposition and not during the non-isothermal cooling stage. This result was also confirmed by a computational simulation of the temperature profile during printing. 

Once the printability of PHBH was demonstrated via rheological analysis, different printing conditions were used to determine a correlation between the printing conditions and the mechanical and morphological properties. The analysis of the dynamic viscoelastic moduli leads to discuss the correlation between the chain entanglement modulus and the welding response of the prints.

Through this study, it was possible to propose that an increase in the temperature of the nozzle from 170 °C to 180 °C (i.e., a temperature below the degradation temperature of PHBH, i.e., 200 °C) and of the bed from 30 °C to 50 °C provokes an improvement of the mechanical properties. In fact, a 15% increase in the tensile strain at break was determined with these printing conditions. Such an improvement is due to the reduction of void formation, as demonstrated by SEM. 

The effect of printing velocity on printing properties was also determined. Printing velocities of 20, 30, and 40 mm/s were used, and intermediate printing velocities resulted in better mechanical properties. This is an advantage in terms of energy savings, as spending more energy printing at higher velocities is not desirable.

It was demonstrated that the mechanical properties are better in the specimens in which the layers are oriented in the same direction with respect to each other and with respect to the test direction. Indeed, there is a 6% increase in Young’s modulus, 17% in tensile strength, and 30% in the strain at break in the samples printed with a raster angle of 90°. Furthermore, the mechanical properties obtained for the 3D-printed specimens are comparable with those obtained from compression molding in terms of stiffness.

## Figures and Tables

**Table 1:** Molecular weight (number-average and weight-average, polydispersity, melt volume rate, and melt flow index of PHBH (data taken from Reference [51]).

M_n_ (kDa)	M_w_ (kDa)	Đ	MVR (cm^3^ 10 min^−1^)	MFI (g 10 min^−1^)
121	163	1.35	18.3	2 (at 165 °C, 5 kg)

**Table 2:** Printing conditions explored in this work.

Name	Nozzle Temperature (°C)	Bed Temperature (°C)	Printing Velocity (mm/s)
L12	180	30	30
L22	180	50	30
M12	190	30	30
M22	190	50	30
M21	190	50	20
M23	190	50	40
H12	200	30	30
H22	200	50	30
M12 [90°, 90°]	190	30	30

**Table 3:** List of parameters of PHBH used in the simulation.

Parameter	Description
Density (*ρ*)in kg/m^3^	1216
Heat capacity (*c_p_*) in J/(kg·°C)	*c_p_*(*T*) = −0.033*T*^2^ + 14.277*T* + 912.36
Thermal conductivity (*k*) in W/(m·°C)	*k*(*T*) = 0.0002*T* + 0.1294 (30 °C < *T* < 95 °C)
	*k*(*T*) = 0.0013*T* + 0.0262 (95 °C < *T* < 105 °C)
	*k*(*T*) = 0.000*Tx* + 0.132 (105 °C < *T* < 105 °C)

**Table 4:** Thermal DSC cooling and heating properties of the PHBH filament.

Sample	Cooling			Second Heating			
	*T_c_* (°C)	Δ*H_c_* (J/g)	*T_cc_* (°C)	Δ*H_cc_* (J/g)	*T_m_* (°C)	Δ*H_m_* (J/g)	*x_c_* (%)
Filament	50.2	43	51.2	17	124.1/143.5	60	49

**Table 5:** Two-domain Tait equation estimated parameters of the PHBH polymer PVT experimental data obtained in isobaric cooling at 5 °C/min.

Solid State		Molten State		Liquid-Solid Transition	
*b*_1*s*_ (cm^3^/g)	0.8540	*b*_1*m*_ (cm^3^/g)	0.9100	*b*_5_ (°C)	98
*b*_2*s*_ (cm^3^/g °C)	4.5 × 10^−4^	*b*_2*m*_ (cm^3^/g °C)	6.628 × 10^−4^	*b*_6_ (°C/Pa^2^)	2.32 × 10^−7^
*b*_3*s*_ (Pa)	1.11910^8^	*b*_3*m*_ (Pa)	6.5970 × 10^7^		
*b*_4*s*_ (°C^−1^)	1.12310^−3^	*b*_4*m*_ (°C^−1^)	1.75 × 10^3^		
*b*_7_ (cm^3^/g)	6.06 × 10^−2^				
*b*_8_ (°C^−1^)	5.4 × 10^−2^				
*b*_9_ (Pa^−1^)	1.9 × 10^−8^				

**Table 6:** Mechanical properties of the 3D pattern printed at different bed and nozzle temperatures.

Condition	Nozzle T (°C)	Bed T (°C)	Printing Velocity (mm/s)	Young Modulus, E (MPa)	Tensile Strength, σ_M_ (MPa)	Strain at Break, ε_B_ (%)
L12	180	30	30	1110 ± 27	14.9 ± 1.0	2.15 ± 0.02
L22	180	50	30	1150 ± 78	17.0 ± 1.3	2.67 ± 0.22
M12	190	30	30	1200 ± 66	16.5 ± 1.4	2.55 ± 0.25
M22	190	50	30	1260 ± 30	18.5 ± 1.4	2.89 ± 0.03
H12	200	30	30	1241 ± 85	16.3 ± 2.0	2.31 ± 0.05
H22	200	50	30	1210 ± 98	17.6 ± 2.6	2.60 ± 0.16

**Table 7:** Mechanical properties of the 3D pattern printed at different printing velocities.

Condition	Nozzle T (°C)	Bed T (°C)	Printing Velocity (mm/s)	Young Modulus, E (MPa)	Tensile Strengh, σ_M_ (MPa)	Strain at Break, ε_B_ (%)
M21	190	50	20	970 ± 90	13.2 ± 3.0	2.53 ± 0.23
M22	190	50	30	1260 ± 30	18.5 ± 1.4	2.89 ± 0.03
M23	190	50	40	1090 ± 105	16.8 ± 1.5	2.91 ± 0.41

**Table 8:** Mechanical properties of the 3D pattern printed at different raster angles compared with the patterns obtained from compression molding.

Condition	Nozzle T (°C)	Bed T (°C)	Printing Velocity (mm/s)	Young Modulus, E (MPa)	Tensile Strength, σ_M_ (MPa)	Strain at Break, ε_B_ (%)
M12 [45°, 45°]	190	30	30	1200 ± 66	16.5 ± 1.4	2.55 ± 0.25
M12 [90°, 90°]	190	30	30	1280 ± 51	20.1 ± 1.0	3.58 ± 0.12
Compression Molding	-	-	-	1320 ± 90	24.0 ± 1.9	6.78 ± 1.0

## Data Availability

Data available upon request.
